# Fabrication of Highly Porous Polymeric Nanocomposite for the Removal of Radioactive U(VI) and Eu(III) Ions from Aqueous Solution

**DOI:** 10.3390/polym12122940

**Published:** 2020-12-09

**Authors:** Tansir Ahamad, Mu. Naushad, Mohd Ubaidullah, Saad Alshehri

**Affiliations:** 1 Department of Chemistry, King Saud University, Riyadh 11451, Saudi Arabia; mnaushad@ksu.edu.sa (M.N.); mtayyab@ksu.edu.sa (M.U.); alshehri@ksu.edu.sa (S.A.); 2 Yonsei Frontier Lab, Yonsei University, Seoul 03722, Korea; 3 School of Life and Allied Health Sciences, Glocal University, Saharanpur 247001, India

**Keywords:** 2,4 dihydroxybenzaldehyde, polymer nanocomposite, radioactive, adsorption

## Abstract

In the present study, a polymeric nanocomposite, CoFe_2_O_4_@DHBF, was fabricated using 2,4 dihydroxybenzaldehyde and formaldehyde in basic medium with CoFe_2_O_4_ nanoparticles. The fabricated nanocomposite was characterized using FTIR, TGA, XRD, SEM, TEM, and XPS analyses. The analytical results revealed that the magnetic nanocomposite was fabricated successfully with high surface area 370.24 m^2^/g. The fabricated CoFe_2_O_4_@DHBF was used as an efficient adsorbent for the adsorption of U(VI) and Eu(III) ions from contaminated water. pH, initial concentration, adsorption time, and the temperature of the contaminated water solution affecting the adsorption ability of the nanocomposites were studied. The batch adsorption results exposed that the adsorption capacity for the removal of U(VI) and Eu(III) was found to be 237.5 and 225.5 mg/g. The adsorption kinetics support that both the metal ions follow second order adsorption kinetics. The adsorption isotherm well fits with the Langmuir adsorption isotherm and the correlation coefficient (R^2^) values were found to be 0.9920 and 0.9913 for the adsorption of U(VI) and Eu(III), respectively. It was noticed that the fabricated nanocomposites show excellent regeneration ability and about 220.1 and 211.3 mg/g adsorption capacity remains with U(VI) and Eu(III) under optimum conditions.

## 1. Introduction

Radioactive substances can be found in the air, water, and soil, polluting the environment. However, the water source can be contaminated using radionuclides, naturally present in rock and soil or released from human activities, such as medical radiology or nuclear power plants [1,2,3]. The long-term exposure of the radionuclides or drinking of contaminated water can cause cancer and other disorders to the human and animals [4,5,6]. The World Health Organization (WHO) considers that 30 μg L^−1^ of Uranium is safe, but a clear no-effect concentration has not been definitively derived yet. The US Environmental Protection Agency (EPA) has adapted this value. Therefore, the development of effective approaches or systems to treat the contaminated water represents an urgent demand for researchers. Several methods, including reverse osmosis, membrane filtration, solvent extraction, electrodialysis, chemical precipitation, and adsorption, have been used for the removal of toxic ions form aqueous solution [7,8,9]. Nevertheless, each technology has advantages and disadvantages especially regarding efficiency and costs (Supplementary Table S1). Among these methods, adsorption is the most effective method because of its high efficiency, low cost, and environmentally friendly nature [10,11,12,13]. However, for adsorption, an efficient adsorbent plays the main role and several adsorbents such as polymer, graphite carbon, clay, metal oxides and nanocomposites have been used for the adsorption of toxic metal ions or organic pollutants from contaminated water [14,15,16]. On the other hand, nowadays, nanotechnology also plays a key role in the adsorption technology and many nanomaterials have been used for the adsorption of organic and inorganic pollutants form aqueous solution [17,18,19,20]. Even so, the poor adsorption capacity and selectivity of these adsorbents reduced their applications for adsorption of radioactive ions [21,22]. Even though the adsorption capacity and selectively of the polymers based adsorbent can be tuned with the functional groups such as carboxylic (-COOH), amines (-NH_2_/-NH) hydroxyl (-OH) and azomethine (C=N-) etc. [23,24,25,26]. Up to now several polymer nanocomposites including polymer/polymer composite, polymer/carbon composite, polymer/clay composite, polymer/ metal oxide composite etc. are the advanced materials and used as adsorbent for the adsorption of several organic and inorganic pollutants from aqueous solution [3,27,28,29,30,31]. However, these polymer nanocomposites challenge with respect their poor, time consuming and expensive separation methods and limits their use at large and industrial scale [32,33,34].Therefore, the fabrication of magnetic polymer nanocomposite with excellent adsorption efficiency and selectivity is an urgent demand of the researchers. The utilization of metal oxide magnetic nanoparticles with polymer matrices provides higher stability, process ability, excellent reusability, and some exciting enhancements caused by the nanoparticle–polymer interface [35,36,37]. Considering these facts, herein, we have prepared a polymer nanocomposite owing to their high adsorption capacity, low cost, magnetism, low toxicity, and reusability. 

The polymeric resin was fabricated using 2,4 dihydroxybenzaldehyde and formaldehyde and its magnetic nanocomposite was prepared with CoFe_2_O_4_ nanoparticles. As-prepared CoFe_2_O_4_@ DHBF was characterized successfully and used for the removal of U(VI) and Eu(III) ions from contaminated water. The batch adsorption techniques were used, changing the pH, initial concentration, adsorbent dose, contact time, and temperature of the solution. The adsorption kinetics, isotherm, and thermodynamics studies were carried out to find out the interaction between the adsorbate and the adsorbents. Moreover, the adsorption mechanisms were fully elucidated by FT-IR and XPS.

## 2. Experimental

### 2.1. Materials 

Briefly, 2,4 dihydroxybenzaldehyde, formaldehyde, cobalt(II)chloride hexahydrate, Iron(III) chloride hexahydrate, NaOH, HCl, ammonia solution, europium(III) chloride hexahydrate were purchased form Sigma Aldrich (Steinheim, Germany). Meanwhile, uranyl acetate dihydrate was purchased form BDH chemicals (Poole, UK). All reactants used were of analytical grade. All the solutions were prepared in deionised water. CoFe_2_O_4_ nanoparticles were prepared according to previously reported method using cobalt(II)chloride and Iron(III) chloride in 1:2 molar ratio using ammonia solution at room temperature [38,39].

### 2.2. Fabrication of the Nanocomposite

The magnetic polymer nanocomposite was fabricated using 2,4 dihydroxybenzaldehyde, formaldehyde under basic condition [40]. In a 200-mL beaker, 2.76 g (0.02 mol) of 2,4 dihydroxybenzaldehyde was dissolved in 10 mL of distilled water and the 6 mL of formaldehyde was added into the solution and was stirred magnetically at room temperature and the pH of the solution was changed to 8 using NaOH solution and then heated at 60 °C for 30 min. After that, 2 g of prefabricated CoFe_2_O_4_ nanoparticles was added and stirred mechanically at 80 °C for 3 h. The resulting mixture was then re-precipitated using methanol and the magnetic nanocomposite was separated magnetically. The fabricated CoFe_2_O_4_@ DHBF was washed, dried, and stored for further used. 

## 3. Results and Discussion

### 3.1. Characterization of the Nanocomposite

**Figure 1:**
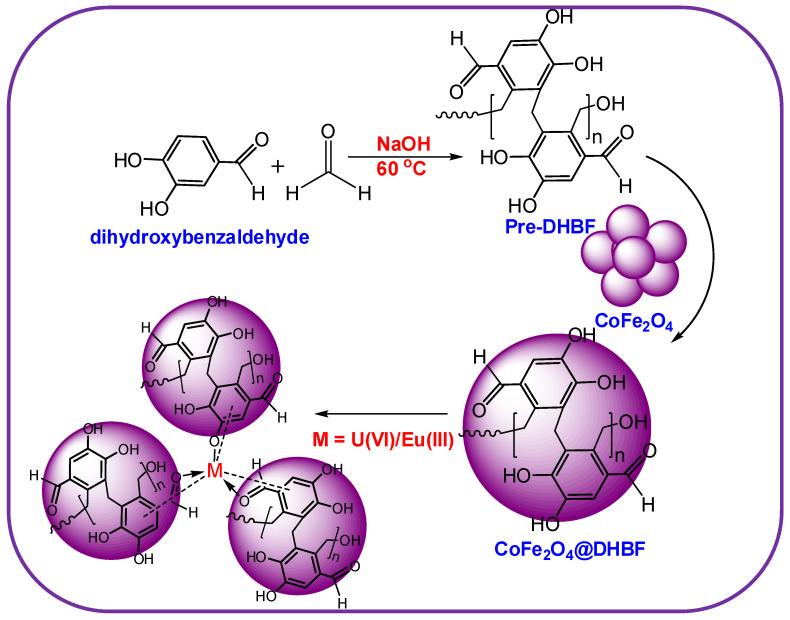
The synthesis routes for the synthesis of CoFe_2_O_4_@DHBF.

The polymers nanocomposite with CoFe_2_O_4_ nanoparticles was prepared using 2,4 dihydroxybenzaldehyde and formaldehyde. The fabrication method is explained in Figure 1. 

**Figure 2:**
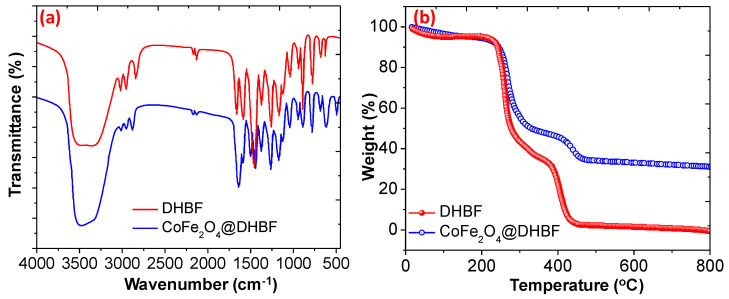
(**a**) FTIR spectra of DHBF and CoFe_2_O_4_@DHBF (**b**) TGA/DTA curves of DHBF and CoFe_2_O_4_@DHBF.

The functional group presents in the polymer and in the nanocomposite were determined using FTIR spectra as shown in Figure 2a. In the case of the dihydroxybenzaldehyde-formaldehyde based polymer resin (DHBF) several FTIR peaks were noticed at 3324–3520 cm^−1^ (O-H), 3044 (C-H aromatic), 2949–2845 (C-H sym and asym), 1663 (C=O), 1564 (C=C), and others [41,42]. Meanwhile, in the case of the magnetic polymer nanocomposite CoFe_2_O_4_@ DHBF the C=O band was shifted from 1163 cm^−1^ to 1649 cm^−1^ was noticed in the presence of CoFe_2_O_4_ nanoparticles and support the interaction between the magnetic nanoparticles and the polymer matrix via hydrogen bonding. Another two FTIR bands were observed at 512 and 627 cm^−1^ and assigned to the Fe-O and Co-O of the spinal cobalt ferrite [43,44]. The TGA was used to investigate the thermal stability and the interaction between the nanoparticles and the polymer matrix in the nanocomposite (CoFe_2_O_4_@ DHBF). The TGA analysis of the polymer and the nanocomposites is shown in Figure 2b. 

**Figure 3:**
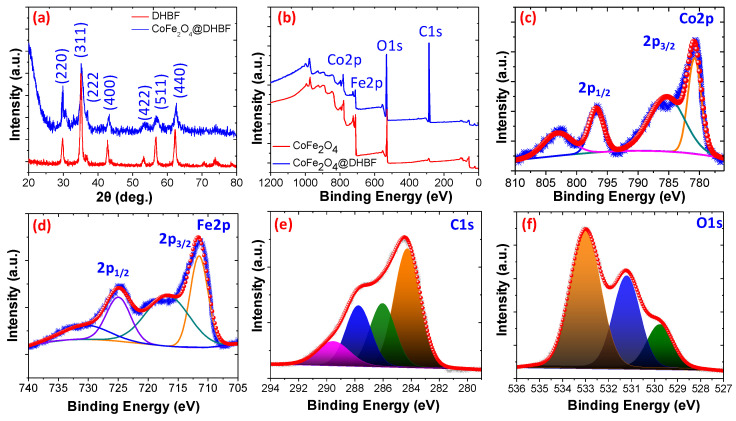
(**a**) XRD of CoFe_2_O_4_, and CoFe_2_O_4_@DHBF (**b**) a wide XPS spectra for CoFe_2_O_4_, and CoFe_2_O_4_@DHBF (**c**) Co2p, (**d**) Fe 2p (**e**) C 1s, (**f**) O1s.

Initially a slightly weight loss about to 4.21% and 5.24% was noticed for DHBF and for CoFe_2_O_4_@DHBF up to 200 °C temperature due to the evaporation of adsorbed humidity and other solvents. Moreover, between 200 to 375 °C about 62.21% and 47.48% weight loss was found and it is the main degradation stage of the organic moieties for DHBF and for CoFe_2_O_4_@ DHBF. The last phase is the cracking of the polymeric materials and at 500 °C about the DHBF decomposed completely while in the case of CoFe_2_O_4_@DHBF about to 13.12% weight loss was notices and the residue weight was found about to 31.20 at 800 °C. These outcomes revealed that the CoFe_2_O_4_@ DHBF shows excellent thermal stability compared to the polymeric resin, DHBF [45,46,47]. The XRD patterns of CoFe_2_O_4,_ and CoFe_2_O_4_@DHBF are illustrated in Figure 3a. It was noticed that the XRD peaks for pure CoFe_2_O_4_ nanoparticles were found at 2θ values 30.14° (220), 35.58° (311), 37.24° (222), 43.34° (400), 53.67° (422), 57.08° (511), 62.7° (440), 71.2° (620), 74.2° (533), 75.2° (622), and 79.2° (444) and can be assigned to the CoFe_2_O_4_ spinel structure (JCPDS no. 22-1086) [48,49]. Moreover, in the case of the CoFe_2_O_4_@DHBF, the intensity of the XRD peaks are decreased without changing their position and the amorphous region peaks also appear. These results support that in the nanocomposites the spinal structure of the CoFe_2_O_4_ is unchanged and embedded it is pure form without any impurity. The X-ray photoelectron spectroscopy (XPS) explained the elemental composition of the CoFe_2_O_4_ and CoFe_2_O_4_@ DHBF. The XPS survey of the CoFe_2_O_4_@DHBF displays the existence of the C, O, Co, and Fe elements as showed in Figure 3b. The deconvoluted spectra of the Co 2p spectra show peaks due to the Co 2p_3/2_ and Co 2p_1/2_ at binding energy of 780.76 and 796.65 eV respectively [50,51]. Meanwhile, the satellites peaks, due to the presence of unpaired 3d electron of the high spin Co^2+^ and belonging to Co 2p_3/2_ and Co 2p_1/2_, appear at a binding energy of 786.19 and 802.95 eV, as shown in Figure 3c. The XPS spectrum of Fe 2p is exposed in Figure 3d and displays two peaks at a binding energy 724.04 and 711.21 eV, assigned to Fe 2p_1/2_ and Fe 2p_3/2_ respectively. These results support the presence of Fe^3+^ in the invers spinel CoFe_2_O_4_. The core-level C1s XPS spectrum is illustrated in Figure 3e and split into four peaks and appeared at binding energy about 283.94, 285.8, 286.80, and 287.72 eV and were assigned to C-C, C=C, C-O, and C=O respectively [52]. The O1s spectrum was split into three peaks and the lattice oxygen appeared at a binding energy of about 529.21, 530.84, and 531.21 eV, Fe-O/Co-O, C-O/C=O, and surface OH, respectively, as illustrated in Figure 3f [53]. 

**Figure 4:**
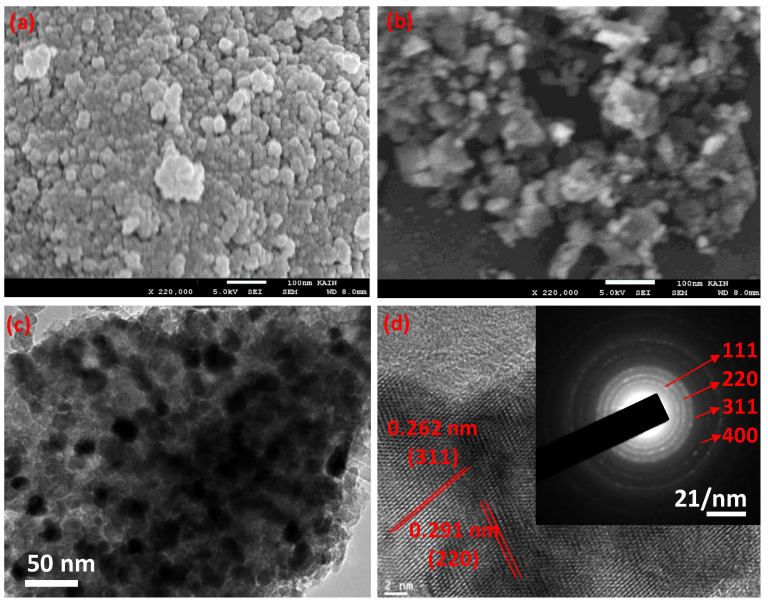
(**a**) SEM image of CoFe_2_O_4_ (**b**) SEM image of CoFe_2_O_4_@DHBF (**c**) TEM image of CoFe_2_O_4_@DHBF (**d**) HRTEM image of CoFe_2_O_4_@DHBF, SAED (inserted).

The surface morphology of the nanoparticles and the nanocomposite was determined using the SEM and TEM analysis. As illustrated in Figure 4a, the SEM image of the CoFe_2_O_4_ shows the spherical shape with a diameter range of 14–25 nm, the fabricated nanoparticles are aggregated due to their super magnetic nature. While in the case of the CoFe_2_O_4_@ DHBF, the CoFe_2_O_4_ nanoparticles are well dispersed into the polymer matrix and no aggregation was noticed. The shape and size of the nanoparticles were unchanged in the case of the nanocomposite. The detailed morphology of the nanocomposite was monitored with a TEM image as shown in Figure 4c and showed similar results to the SEM results. The crystalline nature and the interaction with the polymer matrix were further confirmed with HRTEM analysis and illustrated in Figure 4d. The lattice fingers were noticed with d-spacing of 0.262 and 0.291 nm, which were assigned to the (311) and (220) planes of the CoFe_2_O_4_ spinal structure [54,55,56]. The existence of pure CoFe_2_O_4_ in the polymer nanocomposite was further supported using selected area electron diffraction (SAED) to show the electron diffraction planes as in Figure 4d [49,57]. 

**Figure 5:**
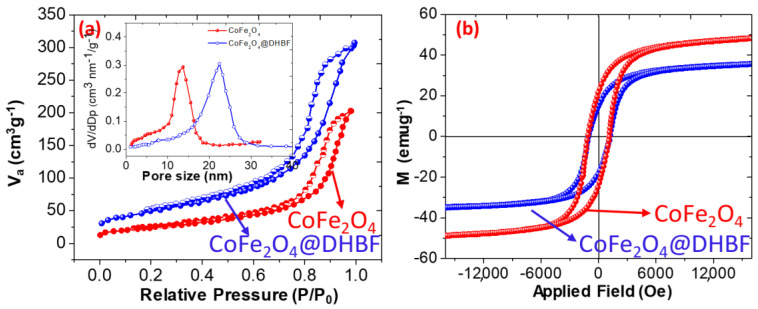
(**a**) N_2_ adsorption and desorption of CoFe_2_O_4_ and CoFe_2_O_4_@DHBF (**b**) magnetic measurements CoFe_2_O_4_ and CoFe_2_O_4_@DHBF.

The N_2_ adsorption and desorption isotherm was used to determine the porous properties of the polymer and the nanocomposite. It was noticed that the adsorption of N_2_ was increased with increasing the relative pressure up to P/P_O_ < 0.8. As shown in Figure 5a, the N_2_ adsorption-desorption shows type IV hysteresis loop and supports the mesoporous nature of the CoFe_2_O_4_@DHBF. The BET results demonstrate that the surface area of the CoFe_2_O_4_ nanoparticles and the CoFe_2_O_4_@DBF was found to be 96.54 and 370.24 m^2^/g respectively. As shown in inserted figure in Figure 5a the pores size were found to be between 12–18 nm and 24–28 nm an indicate both the polymer and the CoFe_2_O_4_@DHBF has mesoporous nature and suitable for the adsorption of the pollutants from aqueous solution. The magnetic behaviors of the pure CoFe_2_O_4_ nanoparticles and the CoFe_2_O_4_@ DHBF were determined using VSM (vibrating-sample magnetometer) analysis and the results were illustrated in Figure 5b. It was observed that the magnetization curves were performed S-shaped with the applied magnetic field and the saturation magnetization (Ms) were found to be 48.50 emu/g and 34.39 emu/g for CoFe_2_O_4_ nanoparticles and the CoFe_2_O_4_@DHBF respectively. In the case of the CoFe_2_O_4_@DHBF, the magnetization was decreased due the nonmagnetic weight ratio of the polymer, DHBF. Hence, the CoFe_2_O_4_@DHBF contains enough magnetization and could be easily and rapidly separated from aqueous solution using a magnet in a very short time. 

### 3.2. Batch Adsorption of the Radioactive Ions 

#### 3.2.1. Effect of pH, Initial Concentration, Initial Contact Time 

**Figure 6:**
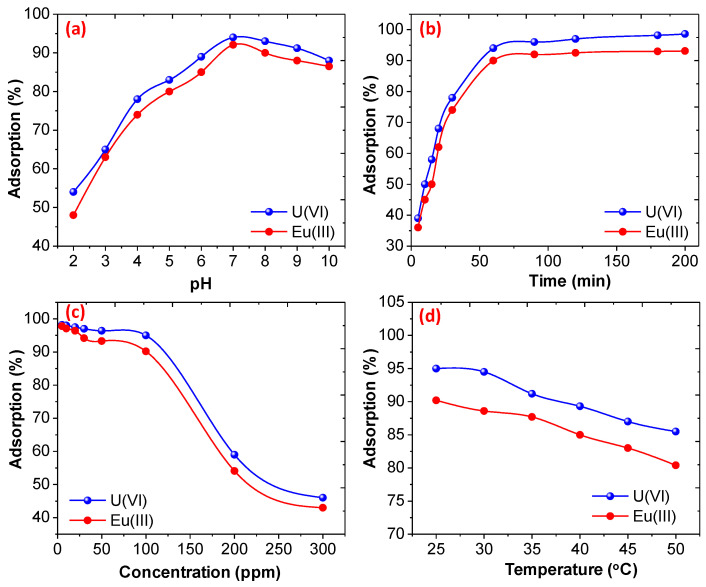
Effect of (**a**) pH (**b**) time (**c**) initial concentration and (**d**) temperature on the adsorption of U(VI) and Eu(III) onto CoFe_2_O_4_@DHBF (dose = 0.01 g, 25 mL, pH = 7, concentration 100 mg/L, time 60 min at room temperature).

The effect of the pH, initial concentration of U(VI) and Eu(III), the dose of the CoFe_2_O_4_@DHBF and the effect of contact time on the removal was thoroughly studied. The pH of the solution is one of the important factor for the removal of the metal ions one CoFe_2_O_4_@DHBF [58]. It was noticed, when the pH of the solution was increased from 2 to 7 the percentage adsorption was increased and found to be 95% and 92.2% (with adsorption capacity 237.5 and 225.5 mg/g) with U(VI) and Eu(III) respectively as display in Figure 6a. Therefore, CoFe_2_O_4_@DHBF has excellent adsorption performance for both the metals, and was far superior most conventional adsorbents listed in Supplementary Table S2. To understand the effect of the pH for the removal of the metal ions pH_pzc_ was determined because the adsorbent surface zeta potential significantly influenced the removal of heavy metal. As shown in Supplementary Figure S1, the pH_pzc_ of the CoFe_2_O_4_@DHBF was found to be 5.13. Therefore, at pH < pH_pzc_ (point of zero charge), the surface charge of the nanocomposite was positive due to extra protons (H^+^), thus the struggle with the metal ions to bind with the adsorption sites, resulting in the active sites of CoFe_2_O_4_@DHBF being protonated and the adsorption capacity for the adsorption of metal ions decreasing due to the presence of extra protons. Moreover, the maximum adsorption was noticed at pH 7. However, when the pH of the solution was further increased, the adoration capacity was decreased due to the formation of the insoluble hydroxide of the corresponding metal ions. The contact time of the adsorbent with the adsorbate affects the adsorption of both the metal ions on CoFe_2_O_4_@DHBF. As shown in Figure 6b, the adsorption of both the metal ions at different time form 5 min to 200 min were studied. It was noticed that the contact time between both U(VI) and Eu(III) increased and the adsorption of both the metal ions increased initially within 30 min, when about 77.9% and 74.0 % of U(VI) and Eu(III) were adsorbed. When the contact time was increased to 60 min, it reaches equilibrium and about 237.5 and 225.5 mg/g adsorption capacity was noticed against the U(VI) and Eu(III) respectively. However, increasing the time further only slightly changed the adsorption capacity. The effects of the initial concentration during the adsorption of the metal ions on to CoFe_2_O_4_@ DHBF was investigated at varying initial concentration from 5–300 mg/L and the results were illustrated in Figure 6c. It was noticed that, when the initial concentration of both the U(VI) and Eu(III) ions was increased, the adsorption percentage of was decreased, while the adsorption capacity of the CoFe_2_O_4_@ DHBF was increased with the initial concentration [59]. Resulting, the initial concentration of 100 mg/L show the highest percentage adsorption and were found to be 237.5 and 225.5 mg/g with U(VI) and Eu(III) respectively within 60 min. Additionally, for the industrial application, the adsorption of both the metal ions at their lower concentration in the range (0.05 to 2 mg/L) were also observed and the results are illustrated in Supplementary Figure S2. The results revealed that the adsorption percentage of both metal ions was increased with decreasing concentration in both the case distilled water and in synthetic wastewater. Moreover, these results revealed that the adsorption of metal ions slightly decreased in the case of the synthetic wastewater sample due to the presence of co-existence ions. 

Initially, the adsorption capacity was increased with the initial concentration. This is because the contact between metal ions and the adsorptive sites of CoFe_2_O_4_@DHBF was increased. However, at high initial concentration, the availability of the adsorptive sites of CoFe_2_O_4_@DHBF were regularly decreased and the saturation took place, resulting in a decreased adsorption capacity with both the metal ions. Moreover, the effects of temperature for the adsorption of both the metal ions were also investigated and the results are displayed in Figure 6d. The adsorption results revealed that the adsorption of both the metal ions was decreased with increasing the temperature of the aqueous solution. Therefore, room temperature is suitable for the adsorption of both the metal ions and was used as an optimum temperature. 

#### 3.2.2. Adsorption Isotherms

**Figure 7:**
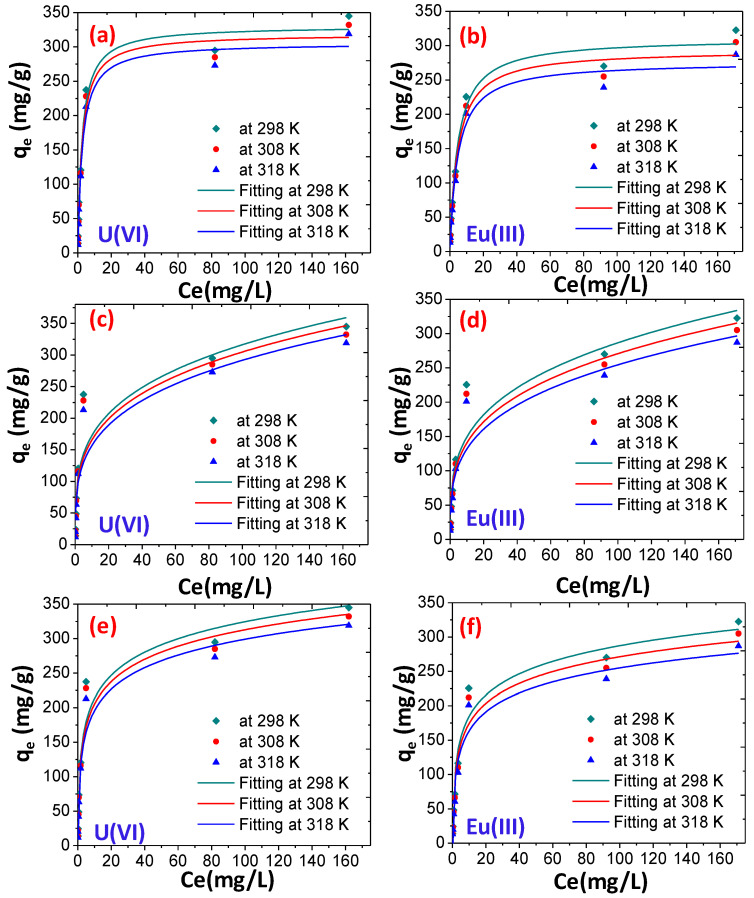
Non-linear fitting for the adsorption of U(VI) and Eu(III) (**a**,**b**) Langmuir (**c**,**d**) Freundlich (**e**,**f**) Temkin isotherm (dose = 0.01 g, 25 mL, pH = 7, time 60 min at room temperature).

To determine the interaction and the adsorption mechanism for the adsorption of U(VI) and Eu(III) onto CoFe_2_O_4_@DHBF, absorption isotherm including Langmuir, Freundlich and Temkin models have been used (the details of adsorption isotherms are given in the Supplementary Materials) [60,61,62]. The nonlinear fittings for these models are displayed in Figure 7, and the results are summarized in Table 1. The results revealed that the experimental data are well fitted with the Langmuir isotherm model and the correlation coefficient (R^2^) values were found to be 0.9920 and 0.9913 for the adsorption of U(VI) and Eu(III), respectively. The calculated adsorption capacity was found to be 330.63 mg/g and 310.70 mg/g with U(VI) and Eu(III) respectively, which were closed to the experimental values at room temperature (298 K). Additionally, the effects of temperature on the adsorption isotherm were also evaluated and the results revealed that, at the increased temperature, the adsorption of both the metal ions was decreased. These outcomes support the fact that adsorption followed the Langmuir isotherm and homogenous monolayers adsorption due to the chemisorption between both the metal ions. 

**Figure 8:**
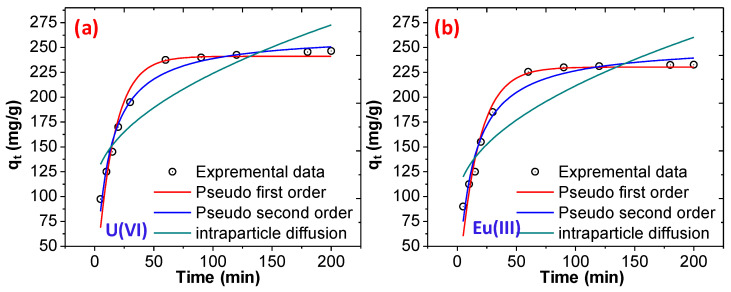
(**a**) Adsorption kinetics for the adsorption of U(VI) (**b**) adsorption kinetics for the adsorption of Eu(III) (dose = 0.01 g, 25 mL, pH = 7, concentration 100 mg/L, at room temperature).

To determine the adsorption rate and adsorption rate constant the adsorption kinetics during the adsorption of U(VI) and Eu(III) on to CoFe_2_O_4_@DHBF was determine using pseudo-first order, pseudo-second order, and interparticle diffusion method and the experimental data was fitted with non-liner model [19,63,64]. The resulting adsorption kinetics parameters were summarized into Table 2. As shown in Figure 8a,b, the pseudo-second order model is well fitted and correlates with the experimental results, the correlation coefficient (R^2^) value was found to be close to 1 (0.9907). Meanwhile, in the case of pseudo-first order and interparticle diffusion, the values of R^2^ were found to be 0.9510 and 0.9514, respectively, during the adsorption of U(VI). The maximum adsorption capacity (q_e_) was determine using pseudo-second order model and found to be 263.89 mg/g and 253.31 mg/g with U(VI) and Eu(III), these values are close to the experimental values. These outcomes support that the adsorption of both the metal ions were follow the pseudo-second order kinetics model and the adsorption capacity of both the metal ions remain constant with time after equilibrium and the available active sites for adsorption depends on the concentration of the metal ions at equilibrium. The adsorption of both the metal ions was chemisorption and the interaction between both the metal ions and the CoFe_2_O_4_@DHBF via coordination of electrons and the covenant.

**Figure 9:**
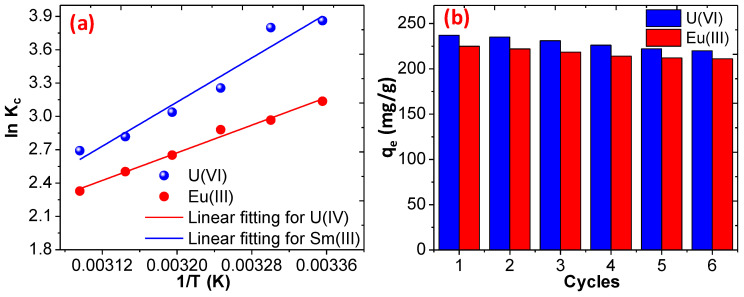
(**a**) adsorption thermodynamic of U(VI) and Eu(III) over CoFe_2_O_4_@DHBF (**b**) regeneration behavior of CoFe_2_O_4_@DHBF (dose = 0.01 g, 25 mL, pH = 7, concentration 100 mg/L, time 60).

The thermodynamics parameters, including change in enthalpy (ΔH), change in entropy (ΔS), and the Gibbs free energy (ΔG), during the adsorption of U(VI) and Eu(III) were determined using the van’t Hoff equation and the details are given in supplementary information [65,66]. The results are illustrated in Figure 9a, and they reveal that the adsorption of both the metal ions proceeds via an exothermic reaction and the negative value of the ΔG supports the spontaneous reaction. The values of ΔG increased with the temperature of the solution and the results are summarized in Table 3. 

### 3.3. Reusability and Regeneration Ability 

The reusability and regeneration of the CoFe_2_O_4_@DHBF was carried out with six cycles and the results are illustrated in Figure 9b. It was noticed that the fabricated nanocomposites show excellent regeneration ability and about to 220.1 and 211.3 mg/g adsorption capacity remains with U(VI) and Eu(III) under optimum conditions (pH = 7, room temperature, initial concentration 100 mg/L, dose of adsorbent 0.01 g, volume 25 mL) [67,68]. The slow decrease in the adsorption capacity with each cycle of regeneration may be due to the loss of the adsorbent during the regeneration because no change in the adsorption site was noticed and supported by the XPS spectra after 3 cycles as shown in Supplementary Figure S3. These outcomes revealed that the CoFe_2_O_4_@DHBF exhibits promising regeneration ability for the adsorption of both the metal ions and in future can be used as a potential adsorbent for the adsorption of toxic pollutants form aqueous solution on an industrial scale.

## 4. Conclusions

Herein, we have fabricated novel nanocomposite and characterized successfully. As-prepared nanocomposite was utilized as capable adsorbent for the removal of U(VI) and Eu(III) form contaminated water. The batch adsorption results exposed that the adsorption capacity for the removal of U(VI) and Eu(III) was found to be 237.5 and 225.5 mg/g, respectively, at room temperature. The optimum condition of the adsorption of both metal ions were pH = 7, initial concentration 100 mg/L, contact time 60 min, and room temperature. The interaction between the metal ions and the CoFe_2_O_4_@DHBF was determine using the adsorption isotherm and adsorption kinetics. The adsorption of both the metals followed the pseudo-second order reaction model and Langmuir adsorption isotherm. The correlation coefficient (R^2^) values of the Langmuir isotherm were found to be 0.9920 and 0.9913 for the adsorption of U(VI) and Eu(III), respectively. Additionally, the reusability results exhibit promising regeneration ability for the adsorption of both the metal ions and in future can be used as a potential adsorbent for the adsorption of toxic pollutants form aqueous solution on industrial scale.

## Figures and Tables

**Table 1:** Adsorption isotherm parameters for the adsorption of U(VI) and Eu(III) on CoFe_2_O_4_@DHBF (dose = 0.01 g, 25 mL, pH = 7, time 60 min at room temperature).

Metal Ions	Isotherm Models	Parameters	Temperature (°C)		
			25	35	45
**U(VI)**	**Langmuir model**	*q*_m_ (mg·g^−1^)	330.63	319.01	3.5.81
		*K*_L_ (L·mg^−1^)	0.3940	0.3879	0.3730
		*R* ^2^	0.9920	0.9929	0.9952
	**Freundlich model**	*K*_f_ (mg^1−1/n^·L^1/n^·g^−1^)	94.24	90.03	89.46
		*n*	3.80	3.77	3.71
		*R* ^2^	0.8670	0.8669	0.8750
	**Temkin** **model**	*K_t_* (L/g)	8.31	8.10	7.76
		*B_t_*	48.27	46.71	44.95
		*R* ^2^	0.9579	0.9487	0.9643
**Eu (III)**	**Langmuir model**	*q*_m_ (mg·g^−1^)	310.70	293.72	276.40
		*K*_L_ (L·mg^−1^)	0.2152	0.2132	0.2113
		*R* ^2^	0.9913	0.9912	0.9901
	**Freundlich model**	*K*_f_ (mg^1−1/n^·L^1/n^·g^−1^)	77.76	73.19	68.34
		*n*	3.53	3.52	3.50
		*R* ^2^	0.8969	0.9010	0.8967
	**Temkin** **model**	*K_t_* (L/g)	6.34	6.32	6.19
		*B_t_*	44.54	42.06	39.67
		*R* ^2^	0.9478	0.9472	0.9439

**Table 2:** Adsorption kinetic parameters for the adsorption of U(VI) and Eu(III) on CoFe_2_O_4_@DHBF.

Metal Ions	Kinetic Models	Parameters	
**U(VI)**	**PFO model**	q_e_ (mg·g^−1^)	241.16
		k_1_ (min^−1^)	0.067
		R^2^	0.9510
	**PSO model**	q_e_ (mg·g^−1^)	263.89
		k_2_ (g·mg^−1^·min^−1^)	3.61 × 10^−4^
		R^2^	0.9907
	**Intra-particle diffusion**	C	106.15
		K_dif_ (mg g^−1^ min^−1/2^)	11.77
		R^2^	0.9514
**Eu(III)**	**PFO model**	q_e_ (mg·g^−1^)	230.31
		k_1_ (min^−1^)	0.060
		R^2^	0.9524
	**PSO model**	q_e_ (mg·g^−1^)	253.31
		k_2_ (g·mg^−1^·min^−1^)	3.30 × 10^−4^
		R^2^	0.9912
	**Intra-particle diffusion**	C	93.58
		K_dif_ (mg g^−1^ min^−1/2^)	11.78
		R^2^	0.8795

**Table 3:** Thermodynamic parameters for the adsorption of U(VI) and Eu(III) on CoFe_2_O_4_@DHBF.

Temperature (K)	U(VI)			Eu(III)		
	Entropy (ΔS)	Enthalpy (ΔH)	Gibbs Free Energy (ΔG)	Entropy (ΔS)	Enthalpy (ΔH)	Gibbs Free Energy (ΔG)
298	−0.0605	−25.87	−7.82	−0.107	−41.79	−9.64
303	−0.0605	−25.87	−7.51	−0.107	−41.79	−9.10
308	−0.0605	−25.87	−7.21	−0.107	−41.79	−8.56
313	−0.0605	−25.87	−6.91	−0.107	−41.79	−8.02
318	−0.0605	−25.87	−6.60	−0.107	−41.79	−7.48
323	−0.0605	−25.87	−6.30	−0.107	−41.79	−6.94

## Supplementary Materials

The following are available online at https://www.mdpi.com/2073-4360/12/12/2940/s1, Table S1: A comparison of removal technologies for the removal of radioactive metal ions, Figure S1: Zeta potential of synthesized CoFe2O4@DHBF, Figure S2: (a) Effect of initial concentration for the adsorption of U(VI) and Eu(III) onto CoFe2O4@DHBF (in distilled water) (b) Effect of initial concentration for the adsorption of U(VI) and Eu(III) onto CoFe2O4@DHBF (in synthetic wastewater) (0.01 g adsorbent, 25 mL, optimum pH = 7, optimum time 60 min at room temperature), Table S2: Comparison of adsorption capacities of Eu(III) and U(VI) by various adsorbents, Figure S3: XPS spectra after adsorption of U(VI) and Eu(III) and after desorption 3 cycles.

## References

[1] Alqadami A.A., Naushad M., Alothman Z.A., Ghfar A.A. (2017). Novel Metal-Organic Framework (MOF) Based Composite Material for the Sequestration of U(VI) and Th(IV) Metal Ions from Aqueous Environment. ACS Appl. Mater. Interfaces.

[2] Asic A., Kurtovic-Kozaric A., Besic L., Mehinovic L., Hasic A., Kozaric M., Hukic M., Marjanovic D. (2017). Chemical toxicity and radioactivity of depleted uranium: The evidence from in vivo and in vitro studies. Environ. Res..

[3] Bai Z.Q., Yuan L.Y., Zhu L., Liu Z.R., Chu S.Q., Zheng L.R., Zhang J., Chai Z.F., Shi W.Q. (2015). Introduction of amino groups into acid-resistant MOFs for enhanced U(vi) sorption. J. Mater. Chem. A.

[4] Chen L., Yu X., Zhao Z. (2007). Effect of humic acid, pH and ionic strength on the sorption of Eu(III) on red earth and its solid component. J. Radioanal. Nucl. Chem..

[5] Dai S., Wang N., Qi C., Wang X., Ma Y., Yang L., Liu X., Huang Q., Nie C., Hu B. (2019). Preparation of core-shell structure Fe_3_O_4_@C@MnO_2_ nanoparticles for efficient elimination of U(VI) and Eu(III) ions. Sci. Total Environ..

[6] Guo W., Nie C., Wang L., Li Z., Zhu L., Zhu L., Zhu Z., Shi W., Yuan L. (2016). Easily prepared and stable functionalized magnetic ordered mesoporous silica for efficient uranium extraction. Sci. China Chem..

[7] Huang S., Pang H., Li L., Jiang S., Wen T., Zhuang L., Hu B., Wang X. (2018). Unexpected ultrafast and high adsorption of U(VI) and Eu(III) from solution using porous Al2O3 microspheres derived from MIL-53. Chem. Eng. J..

[8] Huang Z., Li Z., Zheng L., Zhou L., Chai Z., Wang X., Shi W. (2017). Interaction mechanism of uranium(VI) with three-dimensional graphene oxide-chitosan composite: Insights from batch experiments, IR, XPS, and EXAFS spectroscopy. Chem. Eng. J..

[9] Huang Z.W., Li Z.J., Wu Q.Y., Zheng L.R., Zhou L.M., Chai Z.F., Wang X.L., Shi W.Q. (2018). Simultaneous elimination of cationic uranium(vi) and anionic rhenium(vii) by graphene oxide-poly(ethyleneimine) macrostructures: A batch, XPS, EXAFS, and DFT combined study. Environ. Sci. Nano.

[10] Dao T.-H., Vu T.-Q.-M., Nguyen N.-T., Pham T.-T., Nguyen T.-L., Yusa S.-I., Pham T.-D. (2020). Adsorption Characteristics of Synthesized Polyelectrolytes onto Alumina Nanoparticles and their Application in Antibiotic Removal. Langmuir.

[11] Nguyen N.T., Dao T.H., Truong T.T., Nguyen T.M.T., Pham T.D. (2020). Adsorption characteristic of ciprofloxacin antibiotic onto synthesized alpha alumina nanoparticles with surface modification by polyanion. J. Mol. Liq..

[12] Pham T.D., Bui T.T., Trang Truong T.T., Hoang T.H., Le T.S., Duong V.D., Yamaguchi A., Kobayashi M., Adachi Y. (2020). Adsorption characteristics of beta-lactam cefixime onto nanosilica fabricated from rice HUSK with surface modification by polyelectrolyte. J. Mol. Liq..

[13] Dao T.H., Nguyen N.T., Nguyen M.N., Ngo C.L., Luong N.H., Le D.B., Pham T.D. (2020). Adsorption behavior of polyelectrolyte onto alumina and application in ciprofloxacin removal. Polymers.

[14] Mathews T., Beaugelin-Seiller K., Garnier-Laplace J., Gilbin R., Adam C., Della-Vedova C. (2009). A probabilistic assessment of the chemical and radiological risks of chronic exposure to uranium in freshwater ecosystems. Environ. Sci. Technol..

[15] Ordoñez-Regil E., Ortíz-Oliveros H.B., Fernández-Valverde S.M., Granados-Correa F. (2014). Eu (III) sorption from an aqueous solution onto SrTiO_3_ and surface complex behavior. Chem. Eng. J..

[16] Pham T.D., Tran T.T., Le V.A., Pham T.T., Dao T.H., Le T.S. (2019). Adsorption characteristics of molecular oxytetracycline onto alumina particles: The role of surface modification with an anionic surfactant. J. Mol. Liq..

[17] Ahamad T., Naushad M., Al-Shahrani T., Al-hokbany N., Alshehri S.M. (2020). Preparation of chitosan based magnetic nanocomposite for tetracycline adsorption: Kinetic and thermodynamic studies. Int. J. Biol. Macromol..

[18] Ahamad T., Ruksana, Chaudhary A.A., Naushad M., Alshehri S.M. (2019). Fabrication of MnFe_2_O_4_ nanoparticles embedded chitosan-diphenylureaformaldehyde resin for the removal of tetracycline from aqueous solution. Int. J. Biol. Macromol..

[19] Ahamad T., Naushad M., Eldesoky G.E., Alqadami A.A., Khan A. (2019). Synthesis and characterization of egg-albumen-formaldehyde based magnetic polymeric resin (MPR): Highly efficient adsorbent for Cd(II) ion removal from aqueous medium. J. Mol. Liq..

[20] Naushad M., Alqadami A.A., Ahamad T. (2020). Removal of Cd(II) ion from aqueous environment using triaminotriethoxysilane grafted oxidized activated carbon synthesized via activation and subsequent silanization. Environ. Technol. Innov..

[21] Wang X., Chen L., Wang L., Fan Q., Pan D., Li J., Chi F., Xie Y., Yu S., Xiao C. (2019). Synthesis of novel nanomaterials and their application in efficient removal of radionuclides. Sci. China Chem..

[22] Xie Y., Helvenston E.M., Shuller-Nickles L.C., Powell B.A. (2016). Surface Complexation Modeling of Eu(III) and U(VI) Interactions with Graphene Oxide. Environ. Sci. Technol..

[23] Ghalami Z., Ghoulipour V., Khanchi A. (2019). Highly efficient capturing and adsorption of cesium and strontium ions from aqueous solution by porous organic cage: A combined experimental and theoretical study. Appl. Surf. Sci..

[24] Ogata F., Nagai N., Ueta E., Nakamura T., Kawasaki N. (2018). Biomass potential of virgin and calcined tapioca (cassava starch) for the removal of Sr(II) and Cs(I) from aqueous solutions. Chem. Pharm. Bull..

[25] Bisla V., Rattan G., Singhal S., Kaushik A. (2020). Green and novel adsorbent from rice straw extracted cellulose for efficient adsorption of Hg (II) ions in an aqueous medium. Int. J. Biol. Macromol..

[26] Shi X., Qiao Y., An X., Tian Y., Zhou H. (2020). High-capacity adsorption of Cr(VI) by lignin-based composite: Characterization, performance and mechanism. Int. J. Biol. Macromol..

[27] Barillet S., Adam-Guillermin C., Palluel O., Porcher J.M., Devaux A. (2011). Uranium bioaccumulation and biological disorders induced in zebrafish (Danio rerio) after a depleted uranium waterborne exposure. Environ. Pollut..

[28] Cai Y., Yuan F., Wang X., Sun Z., Chen Y., Liu Z., Wang X., Yang S., Wang S. (2017). Synthesis of core–shell structured Fe_3_O_4_@carboxymethyl cellulose magnetic composite for highly efficient removal of Eu(III). Cellulose.

[29] Chen B., Zhao H., Chen S., Long F., Huang B., Yang B., Pan X. (2019). A magnetically recyclable chitosan composite adsorbent functionalized with EDTA for simultaneous capture of anionic dye and heavy metals in complex wastewater. Chem. Eng. J..

[30] Chen H., Shao D., Li J., Wang X. (2014). The uptake of radionuclides from aqueous solution by poly(amidoxime) modified reduced graphene oxide. Chem. Eng. J..

[31] Chen Z., Xue Z., Chen L., Geng Z., Yang R., Chen L., Wang Z. (2013). One-pot template-free synthesis of water-dispersive Fe_3_O_4_@C nanoparticles for adsorption of bovine serum albumin. New J. Chem..

[32] Carvalho T., Pereira A.d.S., Bonomo R.C.F., Franco M., Finotelli P.V., Amaral P.F.F. (2020). Simple physical adsorption technique to immobilize Yarrowia lipolytica lipase purified by different methods on magnetic nanoparticles: Adsorption isotherms and thermodynamic approach. Int. J. Biol. Macromol..

[33] Dos Santos J.M.N., Pereira C.R., Foletto E.L., Dotto G.L. (2019). Alternative synthesis for ZnFe_2_O_4_/chitosan magnetic particles to remove diclofenac from water by adsorption. Int. J. Biol. Macromol..

[34] Hu D., Lian Z., Xian H., Jiang R., Wang N., Weng Y., Peng X., Wang S., Ouyang X.K. (2020). Adsorption of Pb(II) from aqueous solution by polyacrylic acid grafted magnetic chitosan nanocomposite. Int. J. Biol. Macromol..

[35] Tanhaei B., Ayati A., Iakovleva E., Sillanpää M. (2020). Efficient carbon interlayed magnetic chitosan adsorbent for anionic dye removal: Synthesis, characterization and adsorption study. Int. J. Biol. Macromol..

[36] Yu S., Cui J., Wang J., Zhong C., Wang X., Wang N. (2020). Facile fabrication of Cu(II) coordinated chitosan-based magnetic material for effective adsorption of reactive brilliant red from aqueous solution. Int. J. Biol. Macromol..

[37] Zhang M., Zhang Z., Peng Y., Feng L., Li X., Zhao C., Sarfaraz K. (2020). Novel cationic polymer modified magnetic chitosan beads for efficient adsorption of heavy metals and dyes over a wide pH range. Int. J. Biol. Macromol..

[38] Gabal M.A., Kosa S., Almutairi T.S. (2014). Cr-substitution effect on the structural and magnetic properties of nano-sized NiFe_2_O_4_ prepared via novel chitosan route. J. Magn. Magn. Mater..

[39] Li Z., Liu Y., Zou S., Lu C., Bai H., Mu H., Duan J. (2020). Removal and adsorption mechanism of tetracycline and cefotaxime contaminants in water by NiFe_2_O_4_-COF-chitosan-terephthalaldehyde nanocomposites film. Chem. Eng. J..

[40] Nishat N., Ahmad S., Tansir Ahamad R. (2006). Synthesis and characterization of antibacterial polychelates of urea-formaldehyde resin with Cr(III), Mn(II), Fe(III), Co(II), Ni(II), Cu(II), and Zn(II) metal ions. J. Appl. Polym. Sci..

[41] Jiang R., Zhu H.-Y., Fu Y.-Q., Zong E.-M., Jiang S.-T., Li J.-B., Zhu J.-Q., Zhu Y.-Y. (2020). Magnetic NiFe_2_O_4_/MWCNTs functionalized cellulose bioadsorbent with enhanced adsorption property and rapid separation. Carbohydr. Polym..

[42] Liu F., Zhang W., Chen W., Wang J., Yang Q., Zhu W., Wang J. (2017). One-pot synthesis of NiFe_2_O_4_ integrated with EDTA-derived carbon dots for enhanced removal of tetracycline. Chem. Eng. J..

[43] Deng Y., Zou X., Liu Z., Wang J., Wang Z., Tang J. (2021). Co7Fe_3_/CoFe_2_O_4_@C Lamellar composites derived from Co–Fe LDH/PVA as an effective heterogeneous activator of peroxymonosulfate. J. Alloys Compd..

[44] Gan L., Zhong Q., Geng A., Wang L., Song C., Han S., Cui J., Xu L. (2019). Cellulose derived carbon nanofiber: A promising biochar support to enhance the catalytic performance of CoFe_2_O_4_ in activating peroxymonosulfate for recycled dimethyl phthalate degradation. Sci. Total Environ..

[45] Ahamad T., Nishat N. (2008). New antimicrobial epoxy-resin-bearing Schiff-base metal complexes. J. Appl. Polym. Sci..

[46] Alshehri S.M., Aldalbahi A., Al-Hajji A.B., Chaudhary A.A., in het Panhuis M., Alhokbany N., Ahamad T. (2016). Development of carboxymethyl cellulose-based hydrogel and nanosilver composite as antimicrobial agents for UTI pathogens. Carbohydr. Polym..

[47] Nishat N., Hasnain S., Ahmad T., Parveen A. (2011). Synthesis, characterization, and biological evaluation of new polyester containing Schiff base metal complexes. J. Therm. Anal. Calorim..

[48] Ikram S., Jacob J., Mahmood K., Mehboob K., Maheen M., Ali A., Amin N., Hussain S., Ashraf F., Ilyas S.Z. (2020). A Kinetic study of Tb3+ and Dy3+ co-substituted CoFe_2_O_4_ spinel ferrites using temperature dependent XRD, XPS and SQUID measurements. Ceram. Int..

[49] Kulkarni P., Balkrishna R.G., Ghosh D., Rawat R.S., Medwal R., Chowdari B.V.R., Karim Z., Reddy M.V. (2021). Molten salt synthesis of CoFe_2_O_4_ and its energy storage properties. Mater. Chem. Phys..

[50] Li S., Wu Y., Zheng Y., Jing T., Tian J., Zheng H., Wang N., Nan J., Ma J. (2020). Free-radical and surface electron transfer dominated bisphenol A degradation in system of ozone and peroxydisulfate co-activated by CoFe_2_O_4_-biochar. Appl. Surf. Sci..

[51] Li X., Sun Y., Zong Y., Wei Y., Liu X., Li X., Peng Y., Zheng X. (2020). Size-effect induced cation redistribution on the magnetic properties of well-dispersed CoFe_2_O_4_ nanocrystals. J. Alloys Compd..

[52] Tang J., Wang K., Lu Y., Liang N., Qin X., Tian G., Zhang D., Feng S., Yue H. (2020). Mesoporous core–shell structure NiFe_2_O_4_@polypyrrole micro-rod with efficient electromagnetic wave absorption in C, X, Ku wavebands. J. Magn. Magn. Mater..

[53] Devi R., Gogoi S., Dutta H.S., Bordoloi M., Sanghi S.K., Khan R. (2020). Au/NiFe_2_O_4_ nanoparticle-decorated graphene oxide nanosheets for electrochemical immunosensing of amyloid beta peptide. Nanoscale Adv..

[54] Mansour S.F., Imam N.G., Goda S., Abdo M.A. (2020). Constructive coupling between BiFeO_3_ and CoFe_2_O_4_; promising magnetic and dielectric properties. J. Mater. Res. Technol..

[55] Praveena M.G., Kumar A.S., Kala M.S., Bhowmik R.N., Nair S.S., Thomas S., Anantharaman M.R. (2020). Interface assisted strain-induced magnetoelectric coupling in core-shell nanostructures of CoFe_2_O_4_ @ZnO. J. Magn. Magn. Mater..

[56] Qasim M., Asghar K., Das D. (2019). Preparation and characterization of CoFe_2_O_4_ and CoFe_2_O_4_@Albumen nanoparticles for biomedical applications. Ceram. Int..

[57] Mahdikhah V., Ataie A., Babaei A., Sheibani S., Ow-Yang C.W., Abkenar S.K. (2020). CoFe_2_O_4_/Fe magnetic nanocomposite: Exchange coupling behavior and microwave absorbing property. Ceram. Int..

[58] Ahamad T., Naushad M., Al-Maswari B.M., Ahmed J., Alothman Z.A., Alshehri S.M., Alqadami A.A. (2017). Synthesis of a recyclable mesoporous nanocomposite for efficient removal of toxic Hg2+ from aqueous medium. J. Ind. Eng. Chem..

[59] Ahamad T., Naushad M., Alshehri S.M. (2020). Fabrication of magnetic polymeric resin for the removal of toxic metals from aqueous medium: Kinetics and adsorption mechanisms. J. Water Process Eng..

[60] Alqadami A.A., Naushad M., Alothman Z.A., Ahamad T. (2018). Adsorptive performance of MOF nanocomposite for methylene blue and malachite green dyes: Kinetics, isotherm and mechanism. J. Environ. Manag..

[61] Naushad M., Ahamad T., AlOthman Z.A., Al-Muhtaseb A.a.H. (2019). Green and eco-friendly nanocomposite for the removal of toxic Hg(II) metal ion from aqueous environment: Adsorption kinetics & isotherm modelling. J. Mol. Liq..

[62] Naushad M., Ahamad T., Sharma G., Al-Muhtaseb A.a.H., Albadarin A.B., Alam M.M., Alothman Z.A., Alshehri S.M., Ghfar A.A. (2016). Synthesis and characterization of a new starch/SnO_2_ nanocomposite for efficient adsorption of toxic Hg2+ metal ion. Chem. Eng. J..

[63] Ahamad T., Ruksana, Naushad M., Al-Maswari B.M., Alshehri S.M. (2019). Fabrication of highly porous adsorbent derived from bio-based polymer metal complex for the remediation of water pollutants. J. Clean. Prod..

[64] Alqadami A.A., Naushad M., Abdalla M.A., Ahamad T., Abdullah Alothman Z., Alshehri S.M., Ghfar A.A. (2017). Efficient removal of toxic metal ions from wastewater using a recyclable nanocomposite: A study of adsorption parameters and interaction mechanism. J. Clean. Prod..

[65] Goyal N., Gao P., Wang Z., Cheng S., Ok Y.S., Li G., Liu L. (2020). Nanostructured chitosan/molecular sieve-4A an emergent material for the synergistic adsorption of radioactive major pollutants cesium and strontium. J. Hazard. Mater..

[66] Hu Y., Guo X., Wang J. (2020). Biosorption of Sr2+ and Cs+ onto Undaria pinnatifida: Isothermal titration calorimetry and molecular dynamics simulation. J. Mol. Liq..

[67] Asgari E., Sheikhmohammadi A., Yeganeh J. (2020). Application of the Fe_3_O_4_-chitosan nano-adsorbent for the adsorption of metronidazole from wastewater: Optimization, kinetic, thermodynamic and equilibrium studies. Int. J. Biol. Macromol..

[68] Mohammadabadi S.I., Javanbakht V. (2020). Lignin extraction from barley straw using ultrasound-assisted treatment method for a lignin-based biocomposite preparation with remarkable adsorption capacity for heavy metal. Int. J. Biol. Macromol..

