# Supplementary material for: Fabrication of Highly Porous Polymeric Nanocomposite for the Removal of Radioactive U(VI) and Eu(III) Ions from Aqueous Solution
Source: Polymers (Basel). 2020 Dec 9;12(12):2940. doi: 10.3390/polym12122940 (PMC7763886; doi:10.3390/polym12122940)
Supplement: Supplementary file 1 [file polymers-12-02940-s001.pdf]

Supplementary Materials

Fabrication of Highly Porous Polymeric Nanocomposite for the Removal of Radioactive U(VI) and Eu(III) Ions from Aqueous Solution

Tansir Ahamad [1,*], Mu. Naushad [1,2,3], Mohd Ubaidullah [1] and Saad Alshehri [1]

[1] Department of Chemistry, King Saud University, Riyadh,11451 Saudi Arabia; mnaushad@ksu.edu.sa (M.N.); alshehri@ksu.edu.sa (S.A.); mtayyab@ksu.edu.sa (M.U.)

[2] Yonsei Frontier Lab, Yonsei University, Seoul- 03722 Korea

[3] School of Life and Allied Health Sciences, Glocal University, Saharanpur- 247001, India

* Correspondence: tahamed@ksu.edu.sa

Table S1. A comparison of removal technologies for the removal of radioactive metal ions.

Technology	Advantages	Disadvantages
Chemical precipitation	Relatively low capital cost, simple in operation, common chemicals are available.	Produces toxic sludge. Pre-oxidation may be required, which can produce harmful disinfection by-products. Mainly removes radioactive metal ions. Chemicals dosing, oxidation step, sedimentation and filtration is needed.
Sorption techniques	Relatively well know and commercially available. High removal efficiency. Easy operation and handling. More cost effective. Additional chemical and sludge free. No harmful by-products	Adsorption bed gets exhausted. Periodic replacement of adsorbent material. Yet to be standardized. Produces toxic solid waste.
Ion exchange	Well-defined medium and capacity. The process is less dependent on pH of water. Exclusive ion-specific resin to remove radioactive metal ions.	High cost medium. Requires high-tech operation and maintenance. Regeneration creates a sludge disposal problem. U(VI) is difficult to remove. Exhausted resin regeneration. Applicable for low-TDS. Life of resin
Membrane techniques	Well-defined and high removal efficiency. No toxic solid waste is produced. Capable of removal of other contaminants and microorganisms. Chemicals free.	Very high capital cost. Pre-treatment is needed. High water rejection. High-tech operation and maintenance. Toxic wastewater is produced.
Phytoremediation	Environmental friendly. Chemicals free. Long-term remediation.	Strong developing is needed. No commercially available.
Electro coagulation	Alternative to chemical precipitation. Chemicals free. Novel and promising strategy. Efficient, low cost and easy to maintain.	Strong developing is needed. No commercially available. Focus on effective design and operation parameters is needed.

1. Characterization

The morphologic features of the $CoFe_2O_4$, DHBF and $CoFe_2O_4$@DHBF were observed by field emission scanning electron microscope (FESEM, JEOL, JSM-7600F, Tokyo, Japan) and transmission electron microscopy (TEM, JEOL JEM-2010F, Tokyo, Japan). The typical XRD patterns reflecting the crystal phase structure and purity of the samples were acquired on a Rigaku (Tokyo, Japan) Ultima IV diffractometer over an incident angle from 20° to 80° using Cu-Kα radiation. ATensor-27 (Bruker, Billerica, MA, USA) within the range 400–4000 cm^{-1}, with KBr pellets was used to conduct Fourier transform infrared (FTIR) spectra of as-prepared samples. The magnetic characteristics of the samples

was analyzed by a Lakeshore7404 (Westerville, OH, USA) vibrating sample magnetometer (VSM). The BET specific surface area and pore features were characterized by N_2 adsorption-desorption isotherms conducted on an ASAP 2020 adsorption apparatus (Micromeritics Instrument Co., USA). The surface properties of the photocatalysts was characterized by X-ray photoelectron spectroscopy (XPS) (Thermo ESCALAB 250, USA, Al Kα). Thermogravimetric analysis (TGA) was carried out to check the thermal stability of the resulting materials on using SDTQ 600 (TA Instrument, New Castle, DE, USA) under air atmosphere with 100 mL/min. The zeta potentials of as-proposed $CoFe_2O_4$@DHBF adsorbent at various pH values were measured by Malvern Zetasizer Nano ZS90 (Malvern Instruments Ltd., Malvern, UK).

2. Batch Adsorption Experiments in Single Dye Solution

Batch adsorption experiments in both the metal ion solutions were carried out in duplicate to reveal the adsorption kinetics, isotherms, thermodynamics, effects of initial concentration, effect of temperature, time, and initial solution pH on uptake of U(VI) and Eu(III) onto $CoFe_2O_4$@DHBF. All the experiments were conducted in conical bottles with a capacity of 100 mL by shaking with a speed of 180 rpm in a constant temperature incubator. For all the experiments, 0.01 g of the adsorbent was mixed with 25 mL of metal ions solution with a range of concentration (5–300 mg/L). All the experiments were conducted in conical bottles with capacity of 100 mL by shaking with a speed of 180 rpm in a constant temperature incubator with a range of time form (5–200 min), the initial solution pH was adjusted by using 0.1 M HCl or 0.1 M NaOH. After adsorption, the solid/liquid separation was facilely achieved under an external magnetic field and the residual concentration of target pollutant in aqueous solution was analyzed by inductively coupled plasma atomic emission spectrometry (ICP-AES) and were calculated using flowing Equation (1)[1]:

$$q_e = \frac{(C_0 - C_e)V}{m} \tag{1}$$

where C_0 (mg/L) and C_e (mg/L) represent the initial and equilibrium concentration of U(VI) and Eu(III) respectively. m is the mass of $CoFe_2O_4$@DHBF (g) and V is the volume of U(VI) /Eu(III) solution in L.

3. Synthetic Wastewater

The synthetic wastewater used in the study was prepared using NH_4Cl (1.0 g/L), sodium citrate (0.3 g/L), KH_2PO_4 (0.5 g/L), SO_4^{2-} (1.47 g/L), yeast extract (1.0 g/L), $CaCl_2.2H_2O$ (0.1 g/L), ethylenediaminetetraacetic acid (0.3 g/L), and $FeSO_4.7H_2O$ (0.28 g/L) [2]. Sodium citrate was added in the media to prevent the precipitation of metals in solution prior to the experiments. Sodium lactate (60% v/v) was used as electron donor and the influent sulfate concentration was 1.47 g/L, and for which an equivalent amount of sodium sulfate was added. The U(VI) and Eu(III) ions solutions were prepared with a range of concentration (0.1–2.0 mg/L) using their salts. All reagents used in this study were of analytical grade.

4. Reusability, and Stability of Resultant Adsorbent

To evaluate the potentials of as-resulting adsorbent in practical applications, adsorption-desorption behaviour of U(VI) and Eu(III) by $CoFe_2O_4$@DHBF were systematically checked in present work. After adsorption equilibrium was achieved, the metal ions-loaded adsorbent was magnetically separated and subsequently regenerated by using dil. HCl solution was subject to sonication for 10 min and the concentration of desorbed metal ions was measured by ICP. Afterwards, the regenerated $CoFe_2O_4$@DHBF was washed with copious ultrapure water and reused for the next adsorption process. The above-described adsorption-desorption cycle was consecutively conducted at least three times. Eventually, the reusability as well as stability of the as-prepared adsorbent were evaluated in terms of adsorption capacity.

5. Kinetic Models

Pseudo-first-order, pseudo-second-order, and intra-particle diffusion models were applied to fit the kinetic data in batch adsorption experiments. The mathematical representations of respective models are shown in Equations (2)–(4) [3,4]:

$$q_t = q_e - q_e e^{-k_1 t} \tag{2}$$

$$q_t = \frac{k_2 {q_e}^2 t}{1 + k_2 + q_e t} \tag{3}$$

$$q_t = k_i t^{\frac{1}{2}} + C \tag{4}$$

where q_e (mg g^{-1}) and q_t (mg g^{-1}) are the adsorption capacities at equilibrium and at time t (min), respectively. k_1 (min^{-1}) and k_2 (g mg^{-1}min^{-1}) are the rate constants of the pseudo-first-order model and the pseudo-second-order model, respectively. k_i is intra-particle diffusion rate constant and C is the thickness of the boundary layer.

6. Equilibrium Isotherms

Langmuir, Freundlich, and Temkin isotherm models were used for analysis of the equilibrium data to explore the adsorption essence of U(VI) and Eu(III) on as-designed $CoFe_2O_4$@DHBF adsorbent. The Langmuir isotherm model can be used for the monolayer adsorption process on the adsorbent surface [5]. The nonlinear equation for the Langmuir model is shown below in Equation (5):

$$q_e = \frac{K_L q_{max} C_e}{1 + K_L C_e} \tag{5}$$

where q_e is the amount of metal ions adsorbed on the $CoFe_2O_4$@DHBF (mg/g) and C_e is the aqueous equilibrium concentration of metal ions (mg/L). The dimensionless constant separation factor R_L is given as Equation (6):

$$R_L = \frac{1}{(1 + K_L + C_0)} \tag{6}$$

The R_L parameter denotes the isotherm shape either undesirable (RL > 1), linear (RL = 1), desirable ($0 < R_L < 1$) or irreversible ($R_L = 0$).

The Freundlich adsorption isotherm describes the multilayer adsorption behaviour over the surface of $CoFe_2O_4$@DHBF, the nonlinear Freundlich adsorption isotherm model is described in Equation (7)

$$q_e = K_f C_e^{\frac{1}{n}} \tag{7}$$

where K_f (mg/g (mg/L)$^{-1/n}$) is the Freundlich distribution coefficient, and $1/n$ is empirical constant of the Freundlich describing the adsorption intensity or surface heterogeneity [6]. The Temkin isotherm model contains a factor that explicitly shows adsorbate–adsorbent interactions. It is assumed that the heat of adsorption of all molecules in the layer would decrease with adsorption and the nonlinear model for the Temkin isotherm is given below in Equation (8):

$$q_e = \frac{RT}{b_t} ln k_t \,.\, C \tag{8}$$

where k_t is the Temkin isotherm constant, b_t was related with the maximum bond energy. A b_t value lower than 40 kJ/mol indicates a physical adsorption process. When the b_t is higher than 40 kJ/mol, it directs a chemical adsorption process.

7. Thermodynamic Parameters

It was observed that the adsorption tenancy of U(VI) and Eu(III) over $CoFe_2O_4$@DHBF decreased with with increasing temperature from 296 to 336 K, suggesting that the adsorption metal ions were promoted at lower temperature. Thermodynamic parameters such as free energy (ΔG^o), enthalpy (ΔH^o), and entropy (ΔS^o) change of adsorption were determined by the van't Hoff equation. These parameters are calculated by the following equations:

$$lnK = \frac{-\Delta H^o}{RT} + \frac{\Delta S^o}{R} \quad (9)$$

$$\Delta G^o = \Delta H^o - T\Delta S^o \quad (10)$$

where K is the equilibrium constant, R is the universal gas constant (8.314 J/mol K), and T is the solution temperature (K). The linear plot between lnK and 1/T (K) help to calculate the parameters [7].

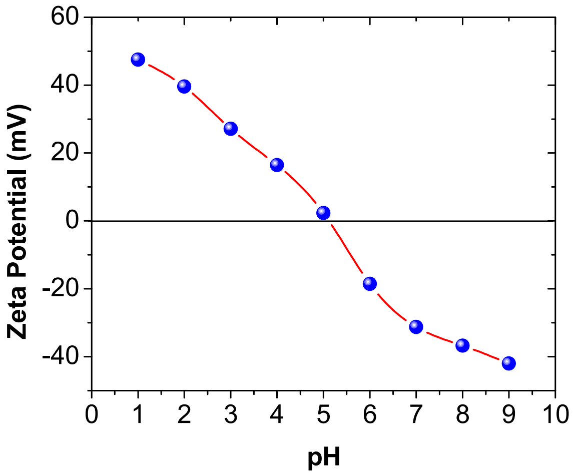

Figure S1. Zeta potential of synthesized $CoFe_2O_4$@DHBF.

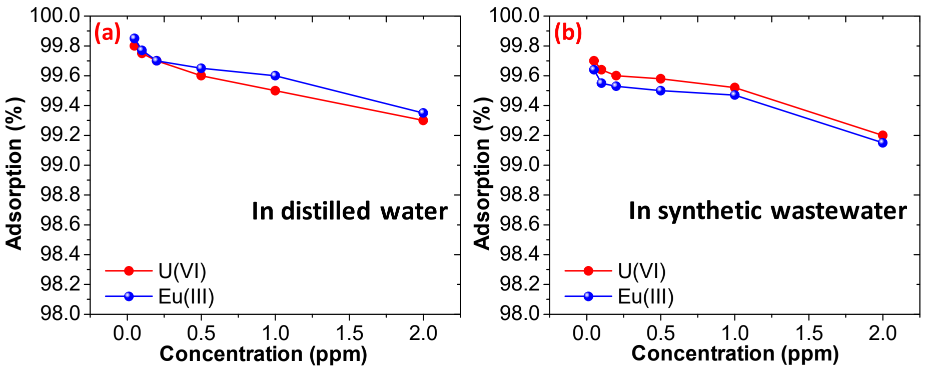

Figure S2. (**a**) Effect of initial concentration for the adsorption of U(VI) and Eu(III) onto $CoFe_2O_4$@DHBF (in distilled water) (**b**) Effect of initial concentration for the adsorption of U(VI) and Eu(III) onto $CoFe_2O_4$@DHBF (in synthetic wastewater) (0.01 g adsorbent, 25 mL, optimum pH = 7, optimum time 60 min at room temperature).

Table S2. Comparison of adsorption capacities of Eu(III) and U(VI) by various adsorbents.

Adsorbents	Metals	Adsorption Capacity (mg/g)	Conditions	References
Activated carbon	Eu(III)	46.5	pH 5	[8]
TiO_2 (P25)	Eu(III)	1.5	pH 4.5	[9]
Molecular sieve	Eu(III)	43.2	pH 5	[10]
Monoclinic pyrrhotite	Eu(III)	10.0	pH 5	[11]
TNTs	Eu(III)	18.7	pH 4.5	[12]
$CoFe_3O_4$@DHBF	Eu(III)	225.5	pH 7	This study
Activated carbon	U(VI)	58.4	pH 5	[13]
Talc	U(VI)	41.6	pH 4	[14]
Natural clinoptilolite zeolite	U(VI)	2.9	pH 6	[15]
Manganese oxide coated zeolite	U(VI)	15.1	pH 4	[16]
TNTs	U(VI)	333	pH 5	[17]
$CoFe_3O_4$@DHBF	U(VI)	237.5	pH 7	This study

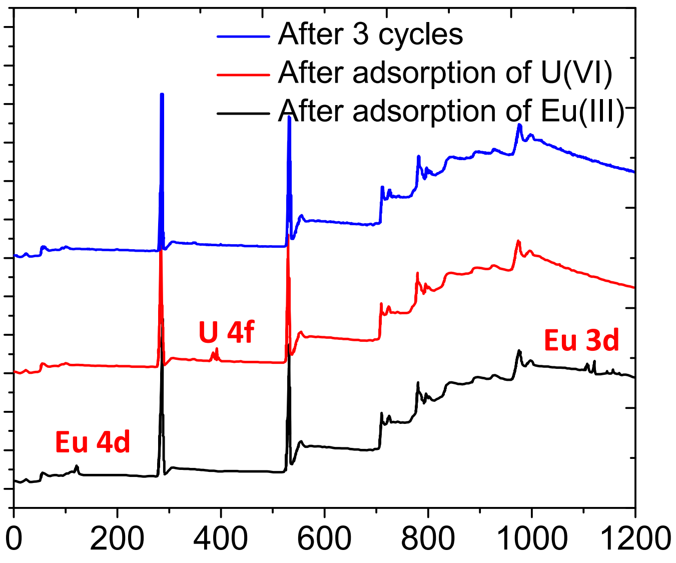

Figure S3. XPS spectra after adsorption of U(VI) and Eu(III) and after desorption 3 cycles.

References.

1. Ahamad, T.; Naushad, M.; Ruksana; Alhabarah, A.N.; Alshehri, S.M. N/S doped highly porous magnetic carbon aerogel derived from sugarcane bagasse cellulose for the removal of bisphenol-A. *Int. J. Biol. Macromol.* **2019**, *132*, 1031–1038.
2. Singanan, M.; Peters, E. Removal of toxic heavy metals from synthetic wastewater using a novel biocarbon technology. *J. Environ. Chem. Eng.* **2013**, *1*, 884–890.
3. Yang, J.; Dai, J.; Wang, L.; Ge, W.; Xie, A.; He, J.; Yan, Y. Ultrahigh adsorption of tetracycline on willow branche-derived porous carbons with tunable pore structure: Isotherm, kinetics, thermodynamic and new mechanism study. *J. Taiwan Inst. Chem. E* **2019**, *96*, 473–482.
4. Okoli, C.P.; Ofomaja, A.E. Degree of time dependency of kinetic coefficient as a function of adsorbate concentration; new insights from adsorption of tetracycline onto monodispersed starch-stabilized magnetic nanocomposite. *J. Environ. Manage.* **2018**, *218*, 139–147.
5. Xie, A.; Cui, J.; Chen, Y.; Lang, J.; Li, C.; Yan, Y.; Dai, J. Simultaneous activation and magnetization toward facile preparation of auricularia-based magnetic porous carbon for efficient removal of tetracycline. *J. Alloys Compd.* **2019**, *784*, 76–87.

6. Islam, M.T.; Hyder, A.H.M.G.; Saenz-Arana, R.; Hernandez, C.; Guinto, T.; Ahsan, M.A.; Alvarado-Tenorio, B.; Noveron, J.C. Removal of methylene blue and tetracycline from water using peanut shell derived adsorbent prepared by sulfuric acid reflux. *J. Environ. Chem. Eng.* **2019**, *7*, 102816.
7. Ahmed, M.J.; Islam, M.A.; Asif, M.; Hameed, B.H. Human hair-derived high surface area porous carbon material for the adsorption isotherm and kinetics of tetracycline antibiotics. *Biores. Technol.* **2017**, *243*, 778–784.
8. Gad, H.; Awwad, N. Factors affecting on the sorption/desorption of Eu (III) using activated carbon. *Separ. Sci. Technol.* **2007**, *42*, 3657–3680.
9. Tan, X.; Fang, M.; Li, J.; Lu, Y.; Wang, X. Adsorption of Eu (III) onto TiO 2: Effect of pH, concentration, ionic strength and soil fulvic acid. *J. Hazard. Mater.* **2009**, *168*, 458–465.
10. Zuo, L.; Yu, S.; Zhou, H.; Jiang, J.; Tian, X. Adsorption of Eu (III) from aqueous solution using mesoporous molecular sieve. *J. Radioanal. Nucl. Chem.* **2011**, *288*, 579–586.
11. Zhu, Y.; Liu, H.; Chen, T.; Xu, B.; Li, P. Kinetics and thermodynamics of Eu (III) adsorption onto synthetic monoclinic pyrrhotite. *J. Mol. Liq.* **2016**, *218*, 565–570.
12. Sheng, G.; Dong, H.; Shen, R.; Li, Y. Microscopic insights into the temperature-dependent adsorption of Eu(III) onto titanate nanotubes studied by FTIR, XPS, XAFS and batch technique. *Chem. Eng. J.* **2013**, *217*, 486–494.
13. Kütahyalı, C.; Eral, M. Selective adsorption of uranium from aqueous solutions using activated carbon prepared from charcoal by chemical activation. *Sep. Purif. Technol.* **2004**, *40*, 109–114.
14. Sprynskyy, M.; Kowalkowski, T.; Tutu, H.; Cukrowska, E.M.; Buszewski, B. Adsorption performance of talc for uranium removal from aqueous solution. *Chem. Eng. J.* **2011**, *171*, 1185–1193.
15. Camacho, L.M.; Deng, S.; Parra, R.R. Uranium removal from groundwater by natural clinoptilolite zeolite: Effects of pH and initial feed concentration. *J. Hazard. Mater.* **2010**, *175*, 393–398.
16. Han, R.; Zou, W.; Wang, Y.; Zhu, L. Removal of uranium (VI) from aqueous solutions by manganese oxide coated zeolite: Discussion of adsorption isotherms and pH effect. *J. Environ. Radioactiv.* **2007**, *93*, 127–143.
17. Liu, W.; Zhao, X.; Wang, T.; Zhao, D.; Ni, J. Adsorption of U(VI) by multilayer titanate nanotubes: Effects of inorganic cations, carbonate and natural organic matter. *Chem. Eng. J.* **2016**, *286*, 427–435.
